# AFM Probing of Amyloid-Beta 42 Dimers and Trimers

**DOI:** 10.3389/fmolb.2020.00069

**Published:** 2020-04-24

**Authors:** Sibaprasad Maity, Yuri L. Lyubchenko

**Affiliations:** Department of Pharmaceutical Sciences, College of Pharmacy, University of Nebraska Medical Center, Omaha, NE, United States

**Keywords:** AFM, amyloid oligomers, force spectroscopy, click chemistry, Aβ42 dimer and trimer, protein-protein interaction

## Abstract

Elucidating the molecular mechanisms in the development of such a devastating neurodegenerative disorder as Alzheimer’s disease (AD) is currently one of the major challenges of molecular medicine. Evidence strongly suggests that the development of AD is due to the accumulation of amyloid β (Aβ) oligomers; therefore, understanding the molecular mechanisms defining the conversion of physiologically important monomers of Aβ proteins into neurotoxic oligomeric species is the key for the development of treatments and preventions of AD. However, these oligomers are unstable and unavailable for structural, physical, and chemical studies. We have recently developed a novel flexible nano array (FNA)-oligomer scaffold approach in which monomers tethered inside a flexible template can assemble spontaneously into oligomers with sizes defined by the number of tethered monomers. The FNA approach was tested on short decamer Aβ(14–23) peptides which were assembled into dimers and trimers. In this paper, we have extended our FNA technique for assembling full-length Aβ42 dimers. The FNA scaffold enabling the self-assembly of Aβ42 dimers from tethered monomeric species has been designed and the assembly of the dimers has been validated by AFM force spectroscopy experiments. Two major parameters of the force spectroscopy probing, the rupture forces and the rupture profiles, were obtained to prove the assembly of Aβ42 dimers. In addition, the FNA-Aβ42 dimers were used to probe Aβ42 trimers in the force spectroscopy experiments with the use of AFM tips functionalized with FNA-Aβ42 dimers and the surface with immobilized Aβ42 monomers. We found that the binding force for the Aβ42 trimer is higher than the dimer (75 ± 7 pN vs. 60 ± 3 pN) and the rupture pattern corresponds to a cooperative dissociation of the trimer. The rupture profiles for the dissociation of the Aβ42 dimers and trimers are proposed. Prospects for further extension of the FNA-based approach for probing of higher order oligomers of Aβ42 proteins are discussed.

## Introduction

Growing evidence has revealed that the neurotoxic effects of diseases such as Alzheimer’s disease (AD) and Parkinson’s disease (PD) result from oligomeric forms of amyloid beta (Aβ) and α-synuclein, respectively (Sengupta et al., 2016; Lee et al., 2017; Ono, 2018). The discovery of highly neurotoxic, small oligomeric species in experimental models for AD and PD (Benilova et al., 2012) highlights the urgent need to explain the properties of these species, considering this knowledge can lead to the development of efficient diagnostic and therapeutic methods. Very limited knowledge exists regarding the structure of these oligomers and molecular mechanisms behind the self-assembly process. The transient nature of these assemblies is a significant challenge in their structural characterization. Considering protein aggregates are stabilized by weak interactions typically transient in nature, they are difficult to measure. Because of these challenges, only mixtures of aggregates with differing morphologies have been studied thus far. In turn, it remains unknown how the aggregation process is initiated and how the growth of oligomers progresses. Teplow group has developed the photo cross-linking method to prepare discrete sizes of Aβ oligomers (Bitan and Teplow, 2004; Ono et al., 2010). These oligomers showed similar neurotoxicity as *in vivo* Aβ oligomers regardless of cross-linking (Ono et al., 2009). Though time-lapse high-speed AFM studies on cross-linked Aβ oligomers (Banerjee et al., 2017b) showed structural dynamics in higher order oligomers (pentamer, hexamer and heptamer), their molecular motions are restricted by cross-linking. Recently, Urbanc and co-workers used a copper and hydrogen peroxide induced cross-linking method for stabilizing Aβ oligomers; however, oligomers remain crosslinked, so drawbacks with the mobility limitations by cross-linking remain (Williams et al., 2016).

Single-molecule approaches are uniquely suitable for tackling the challenge with transient features of amyloid oligomers, and recent reviews outline the progress (Lyubchenko, 2013; Lyubchenko et al., 2016; Ruggeri et al., 2016; Castello et al., 2017; Yang et al., 2018). Optical tweezers were applied in Solanki et al., (2014), enabling the authors to reveal multiple transient states in α-synuclein protein. We developed an AFM-based force spectroscopy method to probe interactions within dimers assembled during interaction of tethered monomers, which was applied to a number amyloidogenic proteins and peptides (Yu et al., 2008, 2011; Yu and Lyubchenko, 2009; Lyubchenko et al., 2010; Kim et al., 2011; Krasnoslobodtsev et al., 2011, 2012, 2013), notably Aβ peptides of various sizes, including full-length Aβ40 and 42 (Kim et al., 2011; Lovas et al., 2013; Lv et al., 2013a, b; Zhang and Lyubchenko, 2014). These studies led to the conclusion that spontaneously assembled dimers are stable and have lifetimes in the range of seconds, which is orders of magnitude larger compared with the characteristic times for the intramolecular dynamics of the monomers (Lyubchenko et al., 2016). The measurements of the stability of amyloid dimers obtained with the AFM dynamics force spectroscopy were in line with direct measurements of dimers’ lifetimes performed with the use of single molecule fluorescence studies (Lv et al., 2015; Maity et al., 2017a). The structures of dimers of Aβ40 and 42 proteins and their segments were revealed with the use of all-atom Molecular Dynamics (MD) simulations (Zhang and Lyubchenko, 2014; Zhang et al., 2016; Hashemi et al., 2019).

AFM force spectroscopy studies revealed novel properties of transiently formed amyloid dimers, this method was limited to probing of dimers. To extend the force spectroscopy technique to oligomers longer than dimers, we developed an approach in which Aβ(14–23) trimers and tetramers were probed with the use of preformed Aβ(14–23) dimers (Maity et al., 2017b). The dimers were assembled using the recently developed flexible nano array (FNA)-oligomer approach in which monomers are tethered inside a flexible polymer, thereby keeping the monomers in close proximity due to the high flexibility of the FNA scaffold (Krasnoslobodtsev et al., 2015; Maity et al., 2016). Using this approach, we were able to assemble dimers, trimers and tetramers of Aβ(14–23) peptides and probe their interactions (Krasnoslobodtsev et al., 2015; Maity et al., 2018a, b).

In this work, we used our FNA method to assemble dimers of the full length Aβ42 protein and measure interactions within the trimer using the AFM force spectroscopy approach with immobilized Aβ42 dimers and monomers. The dimer was assembled spontaneously between the two Aβ42 monomers covalently tethered to the FNA using a metal free click chemistry reaction. Two copies of Aβ42 peptide were attached within the FNA template at selected sites separated by a distance allowing for the spontaneous assembly of the dimer. Flexibility of the FNA segment between the anchoring points was a critical factor for the dimer assembly and was validated by AFM force spectroscopy. To probe the trimer, AFM force spectroscopy experiments were performed with the use of the FNA Aβ42 dimer and Aβ42 monomer. These results reveal elevated stability of Aβ42 trimer compared with the Aβ42 dimer.

## Materials and Methods

Phosphoramidite reagents used for the synthesis of FNA were purchased from Glen Research (VA, United States): spacer 18 phosphoramidite (10–1918), DBCO-dT-CE phosphoramidite (10–1539), Thiol-Modifier C6 S-S (10–1936), biotin CPG (20–2993). N-terminus azide modified Aβ42 [K(N3)-Aβ42] peptide was purchased from GenScript Biotech (NJ, United States). N-(g-Maleimidobutyryloxysuccinimide ester) GMBS was purchased from Pierce Biotechnology (Grand Island, NY). Streptavidin was obtained from Sigma-Aldrich (MO, United States) and Tris-(2-Carboxyethyl) phosphine-HCl (TCEP-HCl) was purchased from Hampton Research Inc. (CA, United States). 1-(3-aminopropyl) silatrane (APS) was synthesized as previously described (Shlyakhtenko et al., 2013). β-mercaptoethanol, NHS-PEG_4_-DBCO and all other reagents or solvents were purchased from Sigma–Aldrich (MO, United States).

### Synthesis of FNA Tether

The polymer was synthesized according to our previous protocol with some modifications (Maity et al., 2016, 2018b). FNA is represented by the following formula indicated from 5′ to 3′: HO-(CH_2_)_6_-S-S-(CH_2_)_6_-(S18)_1_-(DBCO-dT)-(S18)_16_-(DBCO-dT)-(S18)_4_-biotin. The FNA was synthesized in MerMade-12 DNA synthesizer (Bioautomation, United States) using standard protocols, except coupling time of S18 spacer was extented to 10 min. The polymer was synthesized on biotin CPG to generate the following construct: four S18 spacers, DBCO-dT-CE, sixteen S18 spacers, DBCO-dT-CE, one S18 spacer, and a thiol modifier at 5′. DMT group was removed at the end of the synthesis. The product was cleaved from CPG by treating with 30% ammonium hydroxide solution for 16 h, filtered and ammonia was evaporated by vacuum. The crude product was purified by RP-HPLC (solvent A: 0.1 M triethylammonium bicarbonate pH 7.5 buffer, solvent B: acetonitrile, Gradient 20–45% in 40 min, Phenomenex Gemini C18 column, 5 μ, 250 × 4.6 mm). The final purified product was characterized by MALDI-TOF mass spectroscopy.

### Preparation of Monomeric Aβ42 Peptide

Monomeric Aβ42 peptide solution was prepared according to protocol in reference Fezoui et al. (2000). About 0.2 mg of azide Aβ42 peptide was measured on a microbalance (Sartorius AG, Germany), dissolved in 10 mM NaOH solution, and sonicated for 5 min to disrupt preaggregates. The solution was filtered through an amicon spin filter (MWCO 10K) to avoid higher order oligomers, and filtrate was collected. The concentration of peptide was determined from the absorption spectra generated by Nanodrop ND1000 (Thermo-Fisher Scientific, United States) using molar extinction coefficient of tyrosine 1280 M^–1^L cm^–1^ at 280 nm. The peptide solution was then diluted with 10 mM sodium phosphate buffer (pH 7.4) to desired concertation and pH was adjusted to 7.4 if necessary. To check the quality of the sample, AFM imaging was performed that showed only 2–3 small blobs/μm of the surface, which indicates that the majority of molecules are in monomeric state.

### Conjugation of Aβ42 Peptide With FNA

Conjugation of peptide with FNA was performed according the procedure described in Maity et al., (2016), (2018b). FNA contains two DBCO functional groups and two molecules of Aβ42 peptide were conjugated using metal free click reaction. The HPLC purified FNA was dissolved in sodium phosphate buffer (10 mM, pH 7.4) and the concentration was determined from optical absorbance measurement (Nanodrop ND1000, Thermo-Fisher Scientific, United States) considering a molar extinction coefficient of DBCO 12000 M^–1^L cm^–1^at 308 nm (Gong et al., 2016). The FNA solution was diluted to 10 μM final concentration. 60 μM freshly prepared monomeric azide Aβ42 peptide was added and recation was carried out at 4°C to minimize aggregation propencity of peptide. Note that such higher concentartion was used for efficient coupling of Aβ42 to FNA. In this reaction minor products may happen due to coupling of higher oligomers (dimer, trimer or tetramer) to FNA and hence the FNA-Aβ42 monomers was purified by RP-HPLC (solvent A:0.1 M triethylammonium bicarbonate pH 7.5 buffer, solvent B: acetonitrile, Gradient 20–45% in 40 min, Phenomenex Gemini C18 column, 5 μ, 250 × 4.6 mm).

### AFM Tip Functionalization

AFM tips were functionalized with FNA using our protocol described in Maity et al., (2016), (2018b). Briefly, silicon nitride (Si_3_N_4_) AFM tips (MSNL10, Bruker AFM probes, Camarillo, CA) were washed with ethanol and DI water, followed by drying in a gentle flow of argon. Tip surface was treated with UV light (366 nm wavelength) for 45 min and surface was coated with amine functionality by treating the tips with 1 μM APS solution for 30 min, followed by multiple rinses with DI water. To convert amines to maleimide groups, the tips were treated with 1 μM GMBS solution in DMSO for 2 h, followed by multiple rinse steps with DI water. 50 nM Aβ42 conjugated FNA solution was prepared in sodium phosphate buffer (10 mM, pH 7.4) and terminal thiol group was activated by reducing S-S bonds with 10 μM Tris (2-carboxyethyl) phosphine (TCEP). The tips were then immersed into the FNA solution and incubated for 2 h at room temperature. After functionalization, tips were rinsed with DI water and unreacted maleimide groups were quenched by reacting with 10 mM β-mercaptoethanol for 10 min. Finally, the tips were washed with DI water multiple times and stored in 10 mM sodium phosphate buffer (pH 7.4) until used.

### Mica Surface Functionalization With Streptavidin

Mica surface was functionalized with streptavidin by using our previous protocol (Maity et al., 2018a). Briefly, a freshly cleaved mica surface was treated with 167 μM APS solution and incubated for 30 min in a humidified chamber, followed by multiple rinse steps with DI water. The surface was then treated with 0.05% of aqueous glutaraldehyde cross linkers in 10 mM sodium carbonate buffer (pH 7.8) for 30 min and rinsed with DI water. The solution of streptavidin (0.001 μg/mL) was then applied to the surface for 2 h, after which the surface was washed with DI water.

### Mica Surface Functionalization With Aβ42 Monomer

Mica surface was functionalized with Aβ42 monomers according to our previous protocol (Maity and Lyubchenko, 2019). Freshly cleaved mica was coated with amine functionality by treating the surface with 167 μM APS solution for 30 min, followed by multiple rinses with DI water. The surface was covered with 200 μM NHS-PEG_4_-DBCO linker in DMSO, incubated for 1 h, then rinsed with DMSO and DI water. 50 nM monomeric azide Aβ42 peptide (in 10 mM sodium phosphate buffer, pH 7.4) was applied onto the surface and incubated for 2 h. Note that at such low concentration Aβ42 does not aggregate in solution (Banerjee et al., 2017a) and this allowed to obtain low density of peptides on the surface, which is main requirement for single molecule experiments. Finally, the surface was rinsed with DI water and stored at 4°C until used.

### AFM Force Spectroscopy and Data Analysis

Force experiments were performed on Nanowizard 4a Bioscience AFM instrument (JPK, Germany) in 10 mM sodium phosphate buffer, 150 mM NaCl, 1 mM EDTA (pH 7.4) at room temperature. Actual spring constants of AFM cantilevers were determined using the method described by the manufacturer (measured spring constant was in the range of 30–40 pN/nm). For characterization of Aβ42 dimers, the AFM tip functionalized with Aβ42 conjugated FNA was approached to a streptavidin functionalized mica surface. Typically, low trigger force (∼100 pN) was applied toward the surface for 0.5 s to maximize the probability of complex formation. The tip was then retracted at a speed of 500 nm/s. Several thousands of Force-Distance (F-D) curves were acquired to obtain a dataset with several hundred rupture events. Force curves were analyzed with the worm-like chain (WLC) model as described earlier (Krasnoslobodtsev et al., 2015; Maity et al., 2017a, b, 2018b) using the following equation: *F*(x) = k_B_T/Lp[1/4(1−x/Lc)^–2^ − 1/4 + x/Lc], where F(x) is the force at the distance of x, k_B_ is the Boltzmann constant, T is the absolute temperature, and Lp and Lc are the persistence length and contour length, respectively. The data were assembled into histograms and fitted with the Gaussian function to estimate the most probable force and contour length for the specific rupture events. The mean values (maxima in the Gaussian) ± SEM were calculated from the data sets. A similar approach was applied to probing of Aβ42 trimers in which Aβ42 monomer functionalized surface was probed by the AFM tip functionalized by Aβ42-FNA dimers.

## Results

### Experimental Design

**FIGURE 1:**
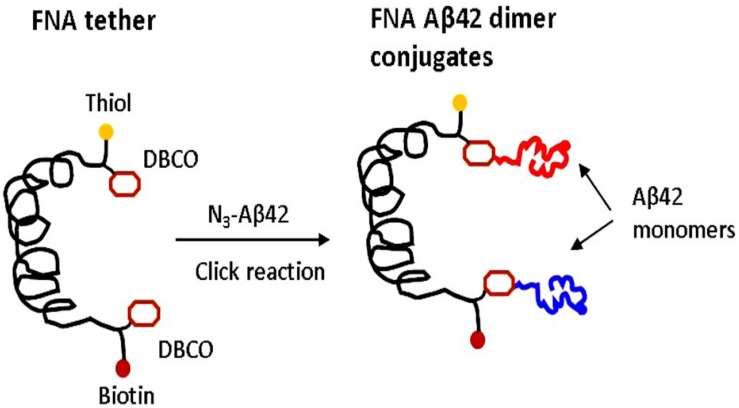
FNA approach for the assembly and probing of Aβ42 dimers. Scheme for the conjugation of the azide group of Aβ42 monomers via DBCO reactive groups incorporated at selected sites of FNA using the metal-free click chemistry. FNA is shown in black. Yellow spheres are thiol groups used for the covalent immobilization of the FNA-Aβ42 dimer on AFM tip. The distance between the DBCO groups is 16 PA units, resulting in a contour length of ∼32 nm for the FNA segments between the anchoring DBCO groups. Red spheres indicate biotin groups. Two Aβ42 monomers are shown in different colors.

The FNA scaffold is a polymer consisting of repeating non-nucleoside phosphoramidite (PA) spacers (Tong et al., 2013). FNA was synthesized using DNA synthesis chemistry, in which PA spacers (18-O-Dimethoxytritylhexaethyleneglycol, 1-[(2-cyanoethyl)-(N,N-diisopropyl)]-phosphoramidite; S18 spacers) were used instead of nucleoside triphosphates. Importantly, each polymerization step was controlled; we used the properties of phosphoramidite chemistry to incorporate non-PA groups at predefined locations to bring reactive sites within FNA (Krasnoslobodtsev et al., 2015; Maity et al., 2016). We used non-metal click chemistry and azide-Aβ42, so two DBCO groups were incorporated as anchor sites for azide-Aβ42. Each PA spacer, along with the groups responsible for the polymerization reaction, contains six polyethylene glycol groups, (PEG)_6_, which provide flexibility for the entire FNA polymeric chain to allow Aβ42 monomers, separated within the FNA by 16 PA units, to assemble into the dimer, as shown in Figure 1. The contour length of the FNA segment with 16 PA units corresponds to the value 32 nm (Tong et al., 2013; Krasnoslobodtsev et al., 2015), so stretching of this segment is sufficient to dissociate the FNA-Aβ42 dimer. The FNA tether contains a terminal thiol group separated from the Aβ anchoring point by one PA unit. The FNA was attached with the AFM probe at this terminal thiol by maleimide-thiol reaction using our developed protocol (Maity et al., 2016, 2018a). The other end of the FNA scaffold separated from another anchoring point for Aβ42 monomer is terminated with biotin and is used in the Aβ42 dimer stretching experiments, in which the surface is functionalized with streptavidin.

### Characterization of Aβ42 Dimer by AFM Force Spectroscopy

**FIGURE 2:**
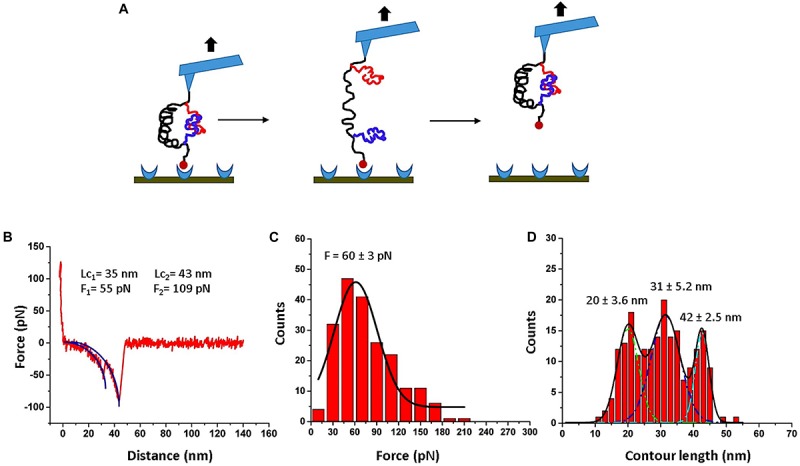
AFM force spectroscopy for FNA-Aβ42 dimer. **(A)** Scheme showing rupture events of the FNA-Aβ42 dimer, followed by dissociation of biotin-streptavidin bonds. **(B)** Representative force curve shows two peaks; first peak corresponds to the dissociation of Aβ42 dimer and the second peak corresponds to the rupture of biotin-streptavidin complex. The blue curve indicates worm-like-chain (WLC) fitting curve. **(C)** The histogram of the rupture force values for the dissociation of the Aβ42 dimer (first peak in B), fitted with a single Gaussian (black line). **(D)** Contour length distribution for the first peak in **(B)** approximated with three Gaussians with the following parameters: 20 ± 3.6 nm, 31 ± 5.2 nm, and 42 ± 2.5 nm. The histograms were obtained by the analysis of 202 rupture events.

To validate the assembly of the dimers, the AFM force spectroscopy set up schematically shown in Figure 2A was used. When the AFM tip is approached to the surface, the FNA construct is captured by the biotin-streptavidin bond, so the retraction step leads to the rupture of the Aβ42 dimer, followed by the dissociation of the biotin-streptavidin bond. This is illustrated by Figure 2A, and a typical rupture curve with two peaks is shown in Figure 2B. Analysis of such force curves was performed in the framework of the WLC model (see section “Materials and Methods”), which resulted in rupture forces of 55 and 109 pN for the first and second peaks, respectively. The first value corresponds well to the rupture force value for Aβ42 dimers obtained in our early force spectroscopy studies (Lv et al., 2013b; Kim and Lyubchenko, 2014), whereas the second peak value is in line with the rupture values of streptavidin-biotin bonds (Teulon et al., 2011; Maity et al., 2018a). This conclusion is supported by a statistical analysis of 202 rupture events out of several thousands of probing events. The data in Figure 2C presents the force distribution for the first peak, and Gaussian approximation yields maxima at 60 ± 3 pN. This force value is consistent with our previous force experiments for the Aβ42 dimer (Lv et al., 2013b; Kim and Lyubchenko, 2014; Zhang et al., 2016), suggesting that Aβ42 monomers tethered inside the FNA scaffold assemble into dimers.

The contour length measurements were performed for the first peaks of force curves. The distribution is shown in Figure 2D. It is broad and was approximated with three Gaussians, yielding peaks at 20 ± 3.6 nm, 31 ± 5.2 nm, and 42 ± 2.5 nm. Similar broad distributions were obtained in the AFM force spectroscopy experiments with Aβ42 monomers, which also revealed three major peaks assigned to interactions between selected segments within the Aβ42 monomers (Lv et al., 2013b; Kim and Lyubchenko, 2014). Computational modeling of the Aβ42 dimer rupture indeed identified these interacting segments, which were assigned to the most strong interactions within the dimers (Zhang et al., 2016). Thus, AFM force spectroscopy studies, along with computer modeling, provided evidence for the assembly of Aβ42 monomers tethered within FNA into dimers.

**FIGURE 3:**
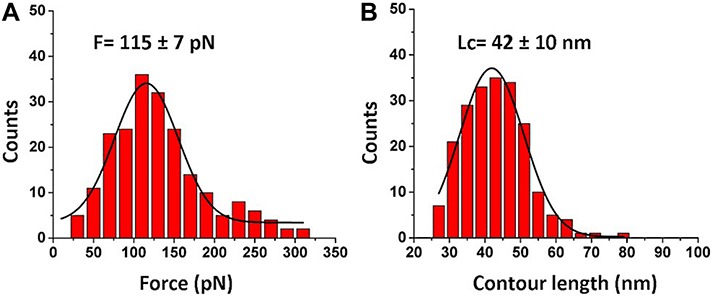
Analysis for biotin-streptavidin rupture events (2^nd^ peak in Figure 2B). **(A)** The histogram for rupture forces. **(B)** The contour length histogram. The Gaussians fits are shown as black curves. The histograms were obtained by the analysis of 202 rupture events.

The force distribution for the second peak is shown in Figure 3A, and the maxima of the Gaussian fitting occurs at 115 ± 7 pN. This value corresponds well to the biotin-streptavidin bonds dissociation strength (Guo et al., 2008; Teulon et al., 2011; Maity et al., 2018a). The contour length distribution for the final rupture event is shown in Figure 3B. The value, 42 ± 10 nm, corresponds to stretching of the entire FNA scaffold. Indeed, FNA has in total 21 PA spacers with 2 nm for each PA spacer (Tong et al., 2013; Lv et al., 2015), so the estimated contour FNA length is 42 nm, which is the same as obtained in the contour length measurements described above.

### Probing of Aβ42 Trimer

**FIGURE 4:**
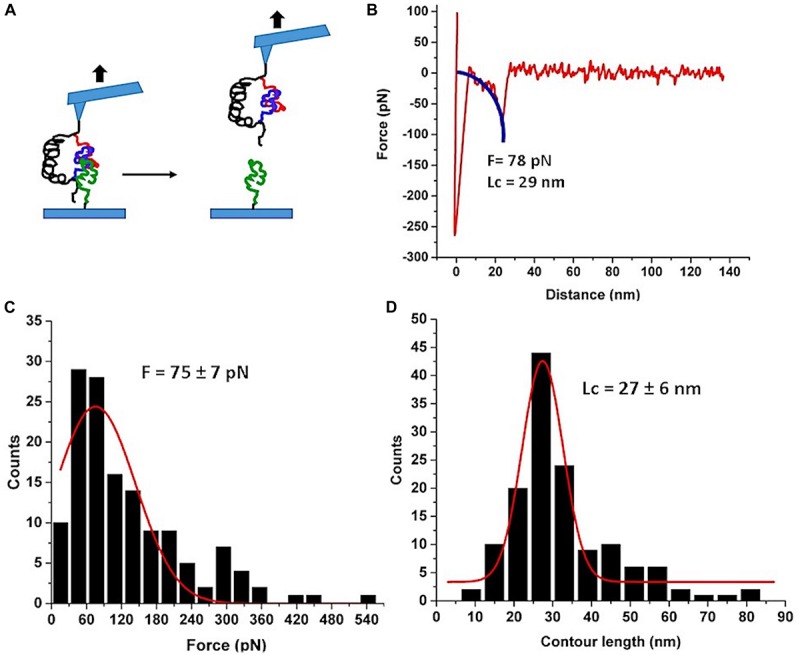
AFM force spectroscopy probing of Aβ42 trimer. **(A)** The schematic for the experimental set up. Three Aβ42 monomers are shown in different colors for clarity. **(B)** A typical force curve of the force spectroscopy experiment. The blue curve is WLC fitting. **(C)** The histogram of the rupture forces approximated with a Gaussian (red curve) with the maximum at 75 ± 7 pN. **(D)** The histogram of contour lengths with the Gaussian fitting. The peak value is 27 ± 6 nm. Number of rupture events used in (**B** and **C**) is 137.

Next, we used the FNA-Aβ42 dimer to probe the trimers using AFM force spectroscopy experiments with immobilized FNA-Aβ42 dimer on the AFM tip and Aβ42 monomer on the mica surface. The experimental setup is shown in Figure 4A. FNA-Aβ42 dimer was immobilized on the AFM tip as described above and the Aβ42 monomer was tethered to the mica surface using a small PEG linker. The trimer was formed when the tip functionalized with FNA-Aβ42 dimer was approached to the surface coated with Aβ42 monomers. The assembly of the trimer is detected by the force curves obtained, with a yield of 6.9%. The majority of force curves show a single peak. A typical force curve is shown in Figure 4B. Similar to the previous analysis, the WLC approximation was applied and values of rupture forces and contour lengths were obtained in these analyses. Figure 4C indicates force distribution for the trimer and Gaussian fitting yields a peak maximum at 75 ± 7 pN. This value is higher than that obtained for the dimer, suggesting that Aβ42 trimers are more stable than dimers. The contour length histogram shown in Figure 4D depicts a narrow distribution approximated with a Gaussian, with the peak at 27 ± 6 nm.

A control experiment was performed, where a surface with no Aβ42 monomers was probed with FNA Aβ42 dimer. The experiment did not produce rupture forces except non-specific adhesions peaks.

## Discussion

### FNA as a Scaffold for the Assembly of Aβ42 Dimer

The results described above demonstrate that the Aβ42 monomers tethered within the FNA scaffold can be assembled into dimers. This was achieved by the use of the FNA scaffold approach, which has a number of key properties enabling a spontaneous self-assembly of Aβ42 monomers into dimers. First, FNA is a flexible polymer, allowing for tethered Aβ42 monomers to move in a broad range necessary for the dimer assembly. Indeed, FNA is synthesized by polymerization of PA units containing six polyethylene glycol groups, so the resulting polymers have a persistence length as small as 0.3 nm, which is very close to the persistence length of PEG polymer (Tong et al., 2013). Second, each polymerization step of the FNA synthesis is controlled, resulting in polymer molecules of identical lengths, which is defined by the FNA synthesis program (Tong et al., 2013). Third, although FNA consisting of PA units is an inert molecule, during the PA chemistry synthesis reactive molecules can be incorporated at selected steps of the synthesis process, and these are the anchoring points for the conjugation of Aβ42 monomers. We used click chemistry to covalently immobilize azide-Aβ42 at dT-DBCO because this coupling reaction has a much higher yield of the product (Wendeln et al., 2012). As a result, these unique properties of the FNA synthesis process allowed us to place two Aβ42 monomers at the distance required for the Aβ42 dimer assembly. The anchoring points for Aβ42 are separated by 16 PA units, which corresponds to ∼32 nm in the contour length (Tong et al., 2013). For the FNA with a persistence length of 0.3 nm (Tong et al., 2013), this segment adopts a random coil conformation, with distances between the anchor points at ∼4 nm, and has a thermal fluctuation in this range (Khokhlov and Grosberg, 1994). According to our MD simulations (Zhang et al., 2016), monomers placed at 4 nm distance assemble into dimers rather rapidly. Indeed, the force spectroscopy data in Figure 2 are in line with the assembly of Aβ42 dimer, and additional analyses as described below support this conclusion.

### Rupture Process of the FNA-Aβ42 Dimer

**FIGURE 5:**
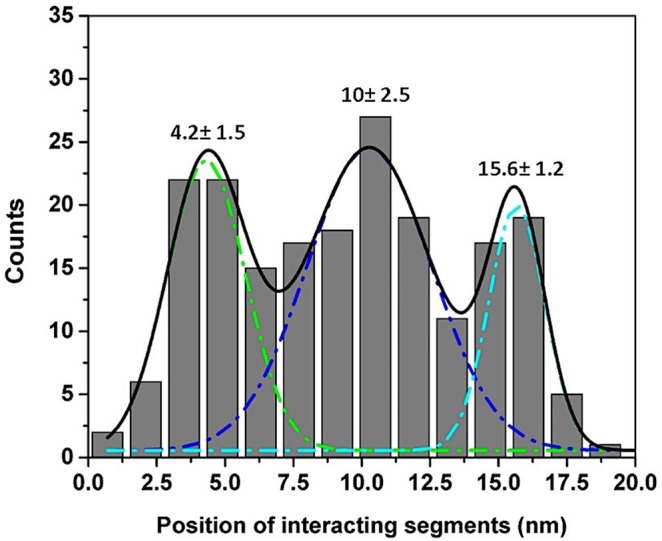
Interacting segments plot for Aβ42 monomer-monomer interactions. The lengths of tethers were subtracted from total Lc and divided by two to obtain locations of interacting segments in each monomer (Lv et al., 2013b). The histogram shows three peaks at 4.2 ± 1.5 nm, 10 ± 2.5 nm, and 15.6 ± 1.2 nm respectively.

**FIGURE 6:**
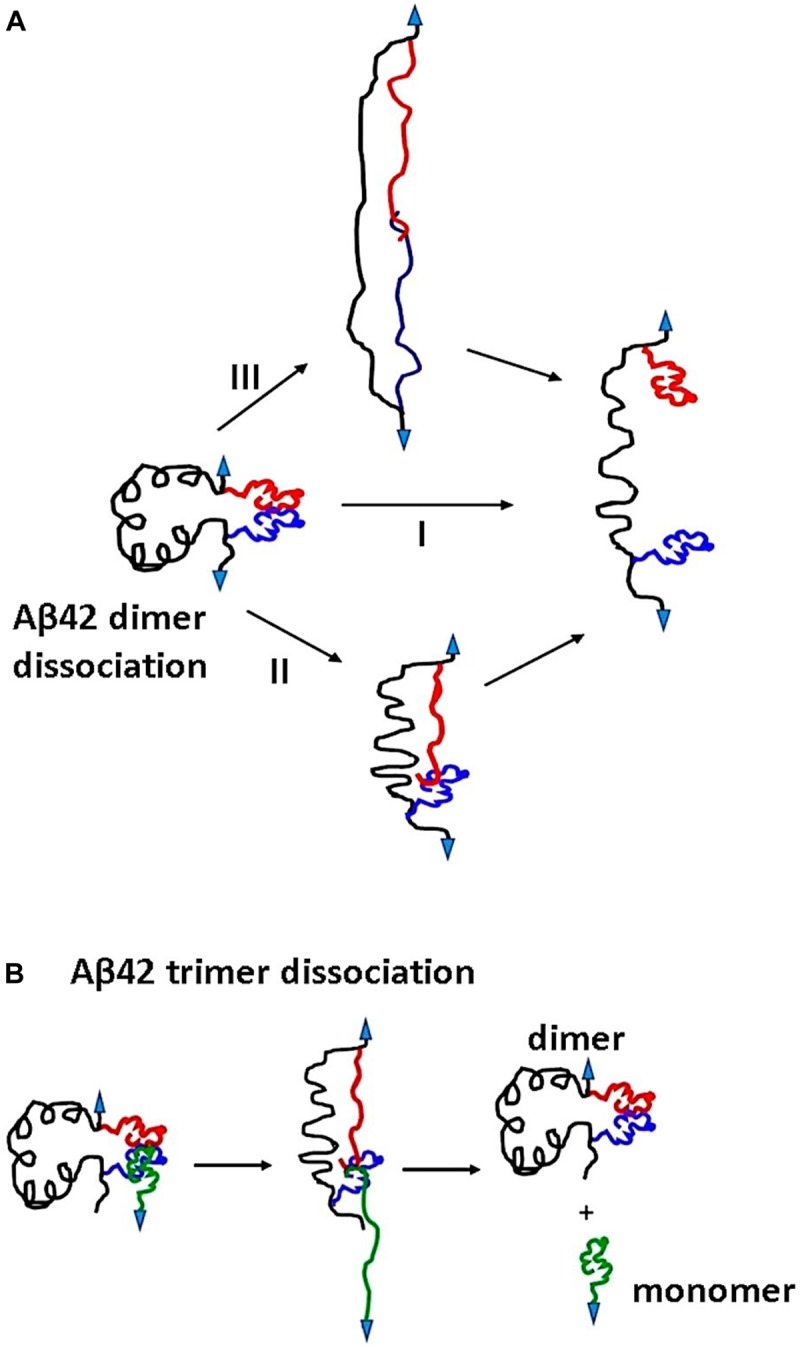
Proposed mechanism for Aβ42 oligomers dissociation pathways. **(A)** Dissociation of Aβ42 dimers occurs in three pathways. Type I, dimers dissociate in their globular forms. Type II involves dimer dissociation through stretching of one monomer although others remain globular. In type III pathway, both the monomers are stretched before dissociation. **(B)** The dissociation pathway for Aβ42 trimer. Three monomers interact at C terminals; during pulling, the surface attached monomer and one of the monomers in the dimer are stretched before dissociation. Arrows indicate point of applied force.

We used the approach described in Lv et al., (2013b) and Maity et al., (2016) to characterize the rupture process of the FNA- Aβ42 dimer. According to Figure 2D, the rupture of two monomers produces three peaks at 20 ± 3.6 nm, 31 ± 5.2 nm, and 42 ± 2.5 nm. Note that as long as biotin-streptavidin rupture force is higher than peptide-peptide interaction, linkers composed of APS, GMBS, and 5 PA spacers (1 PA at AFM tip end + 4 PA at biotin end) were stretched before the Aβ42 dimer dissociation occurs. To determine interaction segments of the peptide, linker length ∼11 nm (APS and GMBS contributed ∼1 nm and 5 PA spacers contributed ∼10 nm) was subtracted from total contour length values of dissociation events, allowing us to identify interacting segments within Aβ42 monomers responsible for the dimer dissociation. Figure 5 demonstrates the distribution of interacting segments lengths, in which three peaks at 4.2 ± 1.5 nm, 10 ± 2.5 nm, and 15.6 ± 1.2 nm are seen clearly. The above results are in line with our previous data on the rupture of the Aβ42 assembled during probing of the tip-immobilized Aβ42 monomer with monomers tethered to the mica surface (Lv et al., 2013b; Maity and Lyubchenko, 2019). Note that computational Monte Carlo Pulling (MCP) modeling of the rupture process for the Aβ42 dimer (Zhang et al., 2016) revealed three distinct pathways for dissociation of Aβ42 dimer, corresponding to three extension distances that are found in these experiments with FNA-Aβ42 dimers. These three dissociation pathways are shown schematically in Figure 6A. In the type I pathway, the dimer dissociates without stretching of monomers, which corresponds to the first peak at 4.2 ± 1.5 nm. Next, the dimer dissociation that occurs by stretching of one monomer while the other is in a collapsed structure, which corresponds to the peak at 10 ± 2.5 nm, is defined by type II pathway. Another potential pathway could occur by the partial stretching of two monomers prior to the dimer dissociation. This option cannot be excluded. However, our MCP simulations are in favor of the asymmetric dissociation model shown in Figure 6A. The peak at 15.6 ± 1.2 nm corresponds to the type III pathway, in which both monomers are fully stretched before the dimer dissociation occurs.

### Rupture Process of Aβ42 Trimer

The availability of FNA-Aβ42 dimer allowed us, for the first time, to probe interactions within Aβ42 trimers assembled during the AFM force probing experiments, as schematically shown in Figure 4A. Compared with the rupture of Aβ42 dimers in these experiments and previous AFM force spectroscopy experiments with Aβ42 monomers (Lv et al., 2013b; Maity and Lyubchenko, 2019) the results for the trimer are different. First, the Aβ42 trimer dissociates at a rupture force of ∼75 ± 7 pN, which is higher than that for the Aβ42 dimers (∼60 ± 3 pN). These data suggest that Aβ42 assembly into trimer further stabilizes the oligomer. This finding is in line with our AFM force spectroscopy experiments for Aβ (14–23) peptides, for which computer modeling experiments revealed sandwich type structures (Maity and Lyubchenko, 2015; Maity et al., 2017a). The elevated stability of the Aβ42 trimer explains why the aggregation process of Aβ42 is shifted toward the assembly of higher order oligomers.

Another striking difference between the Aβ42 dimers and trimers is the rupture profile. According to Figure 4D, the contour length distribution produces one narrow peak with a maxima at 27 ± 6 nm, which is in contrast with the rupture length distribution for Aβ42 dimers that is characterized by three well-separated peaks (Figure 2D). After subtracting the lengths of tethers for the FNA-dimer and the monomer (APS, GMBS, 1 PA spacer for the dimer and four units of PEG for the monomer), which produces a value of ∼4 nm, we obtain a ∼23 nm contour length value, corresponding to stretching of the dimer and monomer prior to the rupture event. This value is considerably less than the loop distance of 32 nm, which is the distance between the monomers within the dimer, suggesting that the rupture of the trimer is not accompanied by the dissociation of the dimer. We obtained a similar contour length value for the type II pathway for the dimer dissociation (Figure 2D). Therefore, we assume that during the trimer rupture, the monomer along with another monomer of the FNA-dimer stretch to produce the contour length value ∼23 nm. Schematically, this dissociation process is shown in Figure 6B. This model suggests that the C-terminal segment of the monomer is involved with the interaction of the FNA-dimer to stabilize the trimer. This model is in line with evidences suggesting that the C-terminal region of Aβ42 is critically involved in aggregation of Aβ42 (Tomaselli et al., 2017). Computer modeling can help to reveal structural features of the trimer dissociation process, and these studies are in progress.

## Conclusion

Overall, we demonstrate here that Aβ42 as dimers can be assembled using the flexible polymeric FNA scaffold, in which two Aβ42 monomers are internally tethered. AFM force spectroscopy data support the dimer assembly with structural and mechanical properties very similar to the ones assembled by non-tethered Aβ42 monomers. Thus, FNA-Aβ42 dimers are available for use with traditional techniques, and we have demonstrated here the use of these dimers for probing the stability of trimers. We found that trimers have elevated stability compared with dimers. As a result, the monomer dissociates from the dimer prior to its dissociation. This finding suggests that interaction of the monomer with the dimer leads to the conformational changes, stabilizing the trimer compared with the dimer. Note that the FNA scaffold can be extended in size, allowing for the tethering of three or more number of monomers, and these studies are in progress. Given that even Aβ42 oligomers as short as dimers are highly neurotoxic, the availability of FNA-Aβ42 dimers open prospects for understanding their disease-prone effects and development of efficient diagnostic and therapeutic treatments for Alzheimer’s disease.

## Data Availability Statement

All datasets generated for this study are included in the article.

## Author Contributions

SM performed the experiments, the data analysis, and the manuscript writing. YL designed the experiments and wrote the manuscript.

## Conflict of Interest

The authors declare that the research was conducted in the absence of any commercial or financial relationships that could be construed as a potential conflict of interest.
